# COVID-19 pandemic-related anxiety, distress and burnout: prevalence and associated factors in healthcare workers of North-West Italy

**DOI:** 10.1192/bjo.2020.161

**Published:** 2021-01-07

**Authors:** Andrea Naldi, Fabrizio Vallelonga, Alessandra Di Liberto, Roberto Cavallo, Monica Agnesone, Marco Gonella, Maria Domenica Sauta, Piergiorgio Lochner, Giacomo Tondo, Nicola Luigi Bragazzi, Rossana Botto, Paolo Leombruni

**Affiliations:** Rita Levi Montalcini Department of Neuroscience, University of Turin, Italy; and Neurology Unit, San Giovanni Bosco Hospital, Italy; Department of Emergency Medicine, San Giovanni Bosco Hospital, Italy; Neurology Unit, San Giovanni Bosco Hospital, Italy; Neurology Unit, San Giovanni Bosco Hospital, Italy; Psychology Unit, Local Health Authority of the City of Turin, Italy; Psychology Unit, Local Health Authority of the City of Turin, Italy; Department of Psychology, University of Turin, Italy; Department of Neurology, Saarland University Medical Center, Germany; School of Psychology, Vita-Salute San Raffaele University, Italy; Department of Mathematics and Statistics, Laboratory for Industrial and Applied Mathematics, York University, Ontario, Canada; Department of Neuroscience, University of Turin, Italy; and Clinical Psychology Unit, City of Health and Science University Hospital of Turin, Italy; Department of Neuroscience, University of Turin, Italy; and Clinical Psychology Unit, City of Health and Science University Hospital of Turin, Italy

**Keywords:** COVID-19, healthcare workers, anxiety, burnout, Italy

## Abstract

**Background:**

The COVID-19 pandemic caused drastic changes in healthcare and severe social restrictions. Healthcare workers (HCWs) are on the front line against the virus and have been highly exposed to pandemic-related stressors, but there are limited data on their psychological involvement for a large sample in Italy.

**Aims:**

To investigate the prevalence of anxiety, distress and burnout in HCWs of North-West Italy during the COVID-19 pandemic, and to detect potential psychosocial factors associated with their emotional response.

**Method:**

This cross-sectional, survey-based study enrolled 797 HCWs. Participants completed the Impact of Event Scale – Revised, the State-Trait Anxiety Inventory – Form Y and the Maslach Burnout Inventory; demographic, family and work characteristics were also collected. Global psychological outcome, differences among professions and independent factors associated with worst psychological outcome were assessed.

**Results:**

Almost a third of the sample had severe state anxiety and distress, high emotional exhaustion and depersonalisation, and low personal accomplishment. Distress was higher in women and nurses, whereas depersonalisation was higher in men. Family division, increased workload, job changes and frequent contact with COVID-19 were associated with worst psychological outcome. Trait anxiety was associated with significantly higher risk for developing state anxiety, distress and burnout.

**Conclusions:**

An elevated psychological burden related to the COVID-19 pandemic was observed in HCWs of North-West Italy. The identification of family and work characteristics and a psychological pre-existing condition as factors associated with worst psychological outcome may help provide a tailored, preventive, organisational and psychological approach in counteracting the psychological effects of future pandemics.

The progressive spread of COVID-19 imposed radical reorganisation in healthcare systems worldwide, to face the ongoing pandemic. Contextually, governments established heavy social restrictions, including quarantine and lockdown, to limit the contagion. The immediate and significant effects of these measures have affected individuals’ quality of life in terms of changes to daily and working routine, as well as limiting their social and affective relationships, possibly leading to a reduction in mental well-being.^1^ Emotional distress during the COVID-19 pandemic has been related to several stressors, including uncertainty regarding health and social and financial repercussions.^2^ A considerable proportion of the general population have suffered anxiety symptoms and an overall increase in depressive disorders, suicidal ideation and trauma-related symptoms has been documented.^3,4^ Healthcare workers (HCWs) are in the front line against the virus, and are highly exposed to the stressors associated with the pandemic. In this extremely demanding health crisis, they have been asked to multiply their efforts through drastic variations in their usual job duties, potentially resulting in an unexpected psychological burden, particularly burnout. Even if burnout syndrome is often described in this occupational category worldwide,^5^ there are limited data available on Italian HCWs during the COVID-19 pandemic, and available data are for small samples.^6,7^ Studies on the psychological impact of previous epidemics on HCWs have shown clinically relevant levels of anxiety, distress and burnout.^8–10^ However, in the modern era, the COVID-19 pandemic is considered to be an unparalleled and incomparable emergency. Preliminary evidence on HCWs exposed to COVID-19 in China and other Eastern countries have confirmed the elevated psychological burden in terms of anxiety, depression, sleep disturbances and post-traumatic symptoms.^11–14^ Nevertheless, although data on the emotional response to the COVID-19 pandemic are increasing, the effects on HCWs of Western nations are understudied and, similarly, little is known about which occupational and socio-familial stressors could have elicited the dysfunctional emotive reactions to the pandemic.

## COVID-19: the situation in Italy during April–May 2020

Northern Italy led the way in the spread of contagion in Europe, and its HCWs were the first in the Western world to experience the dramatic family and occupational rearrangements determined by the pandemic. The first Italian case was isolated on 20 February 2020, and the Italian healthcare system was quickly overwhelmed by the strong rise in positive COVID-19 cases that reached 207,428 on the 1 May 2020, with 28,236 deaths.^15^ The Italian Government imposed quarantine from 10 March to 3 May 2020, corresponding with the highest rate of contagion, hospital admissions and deaths. The implemented restrictive measures were introduced to limit the spread of the virus, and included the suspension of non-essential commercial activities, closure of catering business, a ban on public gatherings, severe limitations to interregional travelling and closure of educational, sociocultural and religious activities.^16^ Despite these restrictions, the progressive and continuous increase in patients with COVID-19 led to the saturation of intensive care units beds and emergency departments in Italian hospitals, followed by significant structural changes and the reorganisation of medical and surgical wards for treatment of patients with COVID-19. As a result, HCWs faced an unexpected increase in workload, busy shifts and changes in their working routine that, combined with the absence of clear guidelines and protocols for the management of the infection, led them to physical exhaustion and feelings of uncertainty, loneliness and alienation.^6^ In addition, the initial unavailability of personal protective equipment and the unpreparedness of the Italian healthcare system massively increased HCWs’ vulnerability and, by 9 April 2020, more than 10,000 cases among HCWs (predominantly in the Northern regions) had been recorded.^17^ Because of direct exposure to potentially infected patients, fear of virus transmission was high, as was the fear of endangering relatives at home. Thus, the use of protective masks was also highly recommended in personal contexts, sometimes determining family division of HCWs to reduce the risk of contagion. All of these factors may have contributed to the development of psychological symptoms in HCWs.

Considering the evidence and the limited data available for Italian HCWs in this area, the aim of this study was to investigate the prevalence of anxiety, distress and burnout in a large sample of physicians and nurses working in Turin city, Piedmont, Northern Italy, during the COVID-19 pandemic, and to detect potential psychosocial family- and work-related factors potentially associated with their emotional response.

## Method

### Study design and participants

This cross-sectional, survey-based study recruited HCWs from four hospitals in Turin city, Piedmont, Northern Italy. The hospitals involved were all facing the pandemic and were active in the management of patients with COVID-19, covering a population area of about 870,000 inhabitants. All procedures contributing to this work comply with the ethical standards of the relevant national and institutional committees on human experimentation and with the Helsinki Declaration of 1975, as revised in 2008. The study received ethical approval by the local institutional review board (A.S.L. Città di Torino). The study was performed according to the Strengthening the Reporting of Observational Studies in Epidemiology Statement (Supplementary material available at https://doi.org/10.1192/bjo.2020.161).

We chose an online survey to collect the data. The online survey allowed for social distancing to be ensured and the elimination of interpersonal interaction, as required by the Italian Government. Before beginning, on the first page of the questionnaire, participants were informed on the scope of the study and their voluntary participation in the survey, which was executed in accordance with current legislation regarding ethical standards and privacy protection. Participants were asked to confirm that they agreed with the information provided, were aged ≥18 years and consented to participation online by selecting the term ‘I agree’. The questionnaire was anonymous and electronic consent was obtained from all participants before taking part. The survey was sent by email simultaneously to all physicians and nurses of the referring hospitals on 27 April 2020, explaining that enrolment would end automatically upon achievement of the target sample size. To create the survey, we used the free platform Google Forms 2020 (Google Inc., USA; see https://www.google.it/intl/it/forms/about/). A minimum sample size of 377 participants was estimated, using the formula *N* = *Z*^2^ × *p*(1−*p*)/*α*^2^, considering a margin of error of 5% (*α* = 0.05), a 95% confidence interval (*Z* = 1.96) and a population proportion of 50% (*P* = 0.50). To favour subgroup analysis, the number of participants was at least doubled. After reaching the target number, recruitment was interrupted at midnight of that day (1 May 2020). These 5 days over which the survey was open were during the more demanding period of the COVID-19 pandemic in Italy (10 March to 3 May 2020). At survey closure, a total of 797 respondents had completed the questionnaire.

### Survey characteristics

The survey investigated the demographic, family and work characteristics of the recruited HCWs, and participants were asked to complete a set of validated rating scales to assess state and trait anxiety, distress and occupational burnout.

Sociodemographic data included age range, gender, job position and the result of a COVID-19 swab test. *Ad hoc* questions on the family situation explored the following areas: having children, current living situation (with partner, without partner) and family division owing to the pandemic (i.e. living separated from own family for safety reasons, fear of endangering relatives). Work queries included contact frequency with patients with COVID-19 patients (‘frequent’ if exposure to infected patients was continuous or almost continuous, ‘rare’ if it was only occasional or absent), occurrence of job changes related to the emergency (i.e. relevant modifications in usual job duties and activities as a result of the pandemic) and variations in workload (increased or not increased, based on the subjective opinion of the HCW).

### Tools

Italian validations of the following three rating scales were used. The Impact of Event Scale – Revised (IES-R)^18,19^ is a self-report scale widely used to measure current subjective distress related to a specific stressful life event, including outbreaks.^20^ It consists of 22 items on a 5-point Likert scale (from 0, ‘not at all’, to 4, ‘extremely’), with respect to how distressing each item has been during the past week. The IES-R assesses symptoms of avoidance, intrusion and hyperarousal. The total score ranges from 0 to 88. The scores identify four levels of distress symptoms: normal, indicating the absence of post-traumatic symptoms (0–23); mild, indicating clinically detectable post-traumatic symptoms (24–32); moderate, indicating the possible presence of post-traumatic stress disorder (33–36); and severe, indicating severe post-traumatic symptoms (≥37).^18^

The State-Trait Anxiety Inventory – Form Y (STAI-Y)^21,22^ is one of the most widely used self-reported measures of anxiety.^23^ It is a self-report inventory aimed at assessing both state (STAI-Y1) and trait (STAI-Y2) anxiety, consisting of 40 items on a 4-point Likert scale (from 1, ‘almost never’, to 4, ‘almost always’).^22^ State anxiety refers to ‘subjective feelings of tension, apprehension, nervousness, and worry, and activation or arousal of the autonomic nervous system, which exist at a given moment in time and at a particular level of intensity’ (page 4).^22^ Trait anxiety refers to ‘relatively stable individual differences in anxiety-proneness, that is, to differences between people in the tendency to perceive stressful situation as dangerous or threatening and to respond to such situations with elevations in the intensity of their state anxiety reactions’ (page 5).^22^ Twenty items are dedicated to each anxiety type. Scores for both subscales range between 20 and 80. For both subscales, scores of 40–50 indicate mild anxiety, 50–60 indicate moderate anxiety and >60 indicate severe anxiety.^22^

The Maslach Burnout Inventory (MBI)^24,25^ is the most common instrument used to measure occupational burnout.^26^ The questionnaire is composed by three scales: emotional exhaustion (nine items), examining the feeling of being emotionally bitter and exhausted from work; depersonalisation (five items), which measures a cold and impersonal response; and personal accomplishment (eight items), assessing the feeling of one's own competence and desire to succeed in working with others. High scores on the personal accomplishment scale, unlike previous emotional exhaustion and depersonalisation scales, indicates greater personal achievement and, consequently, a lower level of burnout. All of the MBI items are scored with a 7-point Likert scale (from 1, ‘never’, to 7, ‘daily’). Consistent with the literature, we considered the level of burnout to be high if emotional exhaustion scores were ≥24, personal accomplishment scores were ≤29 and depersonalisation scores were ≥9; moderate if emotional exhaustion scores were 15–23, personal accomplishment scores were 30–36 and depersonalisation scores were 4–8; and low if emotional exhaustion scores were ≤14, personal accomplishment scores were ≥37 and depersonalisation scores were ≤3.^27^

### Statistical analysis

The normal distribution of variables was tested with Kolmogorov–Smirnov and residual analysis tests. Continuous variables were expressed as mean ± s.d. or median and interquartile range, as appropriate. Qualitative variables were expressed as absolute values of frequency and percentage values. Differences between independent groups were evaluated by a *t*-test for continuous variables with normal distribution, and the Mann–Whitney *U* or Kruskal–Wallis test for continuous variables with non-normal distribution. Categorical variables were compared with the chi-square test or Fisher's exact test, as appropriate. A multivariable logistic regression was applied, using the presence of moderate or severe symptoms (for each psychological scale) as a dependent variable and possible risk factors as independent variables. The associations between risk factors and psychological symptoms are presented as odds ratios and 95% confidence intervals. Statistical significance was considered for *P*-values <0.05 in all analyses. Statistical analysis was performed with software package SPSS (IBM SPSS Statistics for Windows, version 22; IBM Corp., New York, USA).

## Results

### Participants

The study sample was composed of 797 participants (75.2% female); 328 (41.2%) were physicians and 469 (58.8%) were nurses. The majority of the participants (*n* = 528, 66.2%) were aged over 40 years and 563 (70.6%) had frequent contact with patients with COVID-19. A detailed description of the demographics and family and working conditions for both physicians and nurses is provided in Table 1.

**Table 1:** Demographic, family and work characteristics of healthcare workers

		Healthcare workers		
Characteristic	Overall (*n* = 797)	Physician (*n* = 328)	Nurse (*n* = 469)	*P*-value
Gender, *n* (%)				
Male	198 (24.8)	122 (37.2)	76 (16.2)	<0.01
Female	599 (75.2)	206 (62.8)	393 (83.8)	
Age, years, *n* (%)				
18–30	90 (11.3)	18 (5.5)	72 (15.4)	<0.01
31–40	179 (22.5)	83 (25.3)	96 (20.5)	Not significant
41–50	248 (31.1)	90 (27.4)	158 (33.7)	Not significant
>50	280 (35.1)	137 (41.8)	143 (30.5)	<0.01
COVID-19 test, *n* (%)				
Negative	711 (89.2)	300 (91.5)	411 (87.6)	Not significant
Positive	86 (10.8)	28 (8.5)	58 (12.4)	
Children, *n* (%)				
No	291 (36.5)	108 (32.9)	183 (39)	Not significant
Yes	506 (63.5)	220 (67.1)	286 (61)	
Living condition, *n* (%)				
Without partner	233 (29.2)	74 (22.6)	159 (33.9)	<0.01
With partner	564 (70.8)	254 (77.4)	310 (66.1)	
Family division, *n* (%)				
No	591 (74.2)	232 (70.7)	359 (76.5)	Not significant
Yes	206 (25.8)	96 (29.3)	110 (23.5)	
COVID-19 contact, *n* (%)				
Rare	234 (29.4)	99 (30.2)	135 (28.8)	Not significant
Frequent	563 (70.6)	229 (69.8)	334 (71.2)	
Job changes, *n* (%)				
No	349 (43.8)	140 (42.7)	209 (44.6)	Not significant
Yes	448 (56.2)	188 (57.3)	260 (55.4)	
Workload, *n* (%)				
Not increased	322 (40.4)	164 (50)	158 (33.7)	<0.01
Increased	475 (59.6)	164 (50)	311 (66.3)	

### Psychological outcomes

A total of 618 (77.5%) participants had state anxiety and 478 (60%) reported distress symptoms such as avoidance, intrusion and hyperarousal. A considerable proportion of these HCWs suffered severe symptoms, both in terms of state anxiety (*n* = 186, 23.3%) and distress (*n* = 286, 35.9%).

Regarding the three dimensions of occupational burnout, 324 (40.7%) HCWs showed high emotional exhaustion, 241 (30.2%) displayed high depersonalisation and 290 (36.4%) had low personal accomplishment (see Table 2 for full details). Data on trait anxiety, including scores and levels, are reported in the Supplementary Material.

**Table 2:** Psychological outcomes stratified for scores and severity categories

Psychological scale	Overall (*n*=797)	Healthcare workers		*P*-value
		Physician (*n* = 328)	Nurse (*n* = 469)	
STAI Y1					
Total scoring, median (IQR)		50 (41–59)	49 (40–58)	50 (41–60)	Not significant
Categories, *n* (%)	Normal	179 (22.5)	74 (22.6)	105 (22.4)	Not significant
	Mild	218 (27.4)	92 (28)	126 (26.9)	
	Moderate	214 (26.9)	98 (29.9)	116 (24.7)	
	Severe	186 (23.3)	64 (19.5)	122 (26)	
IES-R					
Total scoring, median (IQR)		29 (17–44)	26 (15–41)	31 (20–46)	0.01
Hyperarousal		1.29 (0.7–1.9)	1.14 (0.7–1.9)	1.29 (0.7–2)	Not significant
Avoidance		0.75 (0.3–1.5)	0.75 (0.3–1.5)	1 (0.5–1.5)	<0.01
Intrusion		1.75 (1–2.5)	1.5 (1–2.3)	2 (1.3–2.8)	<0.01
Categories, *n* (%)	Normal	319 (40)	151 (46)*	168 (35.8)*	0.03
	Mild	128 (16.1)	49 (14.9)	79 (16.8)	
	Moderate	64 (8)	25 (7.6)	39 (8.3)	
	Severe	286 (35.9)	103 (31.3)*	183 (39)*	
MBI – emotional exhaustion					
Total scoring, median (IQR)		20 (10–31)	20 (10–33)	19 (10–30)	Not significant
Categories, *n* (%)	Low	297 (37.3)	121 (36.9)	176 (37.5)	Not significant
	Medium	176 (22.1)	70 (21.3)	106 (22.6)	
	High	324 (40.7)	137 (41.8)	187 (39.9)	
MBI – depersonalisation					
Total scoring, median (IQR)		5 (1–10)	5 (2–10)	4 (1–10)	Not significant
Categories, *n* (%)	Low	346 (43.4)	128 (39)	218 (46.5)	Not significant
	Medium	210 (26.3)	93 (28.4)	117 (24.9)	
	High	241 (30.2)	107 (32.6)	134 (28.6)	
MBI – personal accomplishment					
Total scoring, median (IQR)		34 (26–39)	34 (26–39)	34 (26–40)	Not significant
Categories, *n* (%)	Low	290 (36.4)	113 (34.5)	177 (37.7)	Not significant
	Medium	222 (27.9)	98 (29.9)	124 (26.4)	
	High	285 (35.8)	117 (35.7)	168 (35.8)	

STAI Y1, State-Trait Anxiety Inventory Form Y1; IQR, interquartile range; IES-R, Impact of Event Scale – Revised; MBI, Maslach Burnout Inventory.

* Statistically significant.

By stratifying for symptom severity levels, no differences were found between physicians and nurses for either anxiety or occupational burnout. Conversely, nurses showed a higher prevalence of severe distress than physicians (183 [39%] *v*. 103 [31.3%], *P* = 0.03; see Table 2).

### Factors associated with burnout, anxiety and distress

A complete summary of how the demographic, family and occupational factors are associated with each psychological scale is provided in Tables 3 and 4. With regard to gender, women reported higher levels of severe state anxiety and distress than men (26.2% *v*. 14.6%, *P* < 0.01 and 37.9% *v*. 29.8%, *P* = 0.01, respectively), whereas men had a higher level of depersonalisation than women (38.9% *v*. 27.4%, *P* < 0.01).

**Table 3:** Severity categories of evaluated psychological outcomes: subgroups analysis

	STAI Y1 (category)					IES-R (category)				
Characteristic	Normal	Mild	Moderate	Severe	*P*-value	Normal	Mild	Moderate	Severe	*P-*value
Overall, *n* (%)	179 (22.5)	218 (27.4)	214 (26.9)	186 (23.3)	–	319 (40)	128 (16.1)	64 (8)	286 (35.9)	–
Profession, *n* (%)										
Physician	74 (22.6)	92 (28)	98 (29.9)	64 (19.5)	Not significant	151 (46)*	49 (14.9)	25 (7.6)	103 (31.4)*	0.03
Nurse	105 (22.4)	126 (26.9)	116 (24.7)	122 (26)		168 (35.8)*	79 (16.8)	39 (8.3)	183 (39)*	
Gender, *n* (%)										
Male	63 (31.8)*	60 (30.3)	46 (23.2)	29 (14.6)*	<0.01	100 (50.5)*	28 (14.1)	11 (5.6)	59 (29.8)*	0.01
Female	116 (19.4)*	158 (26.4)	168 (28)	157 (26.2)*		219 (36.6)*	100 (16.7)	53 (8.8)	227 (37.9)*	
COVID-19 test, *n* (%)										
Negative	156 (21.9)	202 (28.4)	193 (27.1)	160 (22.5)	Not significant	290 (40.8)	109 (15.3)	57 (8)	255 (35.9)	Not significant
Positive	23 (26.7)	16 (28.6)	21 (24.4)	26 (30.2)		29 (33.7)	19 (22.1)	7 (8.1)	31 (36)	
Children, *n* (%)										
Yes	110 (21.7)	143 (28.3)	145 (28.7)	108 (21.3)	Not significant	205 (40.5)	74 (14.6)	41 (8.1)	186 (36.8)	Not significant
No	69 (23.7)	75 (25.8)	69 (23.7)	78 (26.8)		114 (39.2)	54 (18.6)	23 (7.9)	100 (34.4)	
Living condition, *n* (%)										
With partner	129 (22.9)	159 (28.2)	149 (26.4)	127 (22.5)	Not significant	226 (40.1)	90 (16)	41 (7.3)	207 (36.7)	Not significant
Without partner	50 (21.5)	59 (25.3)	65 (27.9)	59 (25.3)		93 (39.9)	38 (16.3)	23 (9.9)	79 (33.9)	
Family division, *n* (%)										
Yes	30 (14.6)*	48 (23.3)	76 (36.9)*	52 (25.2)	<0.01	49 (23.8)*	37 (18)	15 (7.3)	105 (51)*	<0.01
No	149 (25.2)*	170 (28.8)	138 (23.4)*	134 (22.7)		270 (45.7)*	91 (15.4)	49 (8.3)	181 (30.6)*	
COVID-19 contact, *n* (%)										
Rare	61 (26.1)	65 (27.8)	53 (22.6)	*55* (*23.5)*	Not significant	123 (52.6)*	34 (14.5)	17 (7.3)	60 (25.6)*	<0.01
Frequent	118 (21)	153 (27.2)	161 (28.6)	*131* (*23.3)*		196 (34.8)*	94 (16.7)	47 (8.3)	226 (40.1)*	
Job changes, *n* (%)										
No	93 (26.6)	95 (27.2)	89 (35.5)	*72* (*20.6)*	Not significant	153 (43.8)	67 (19.2)	24 (6.9)	105 (30.1)*	0.01
Yes	86 (19.2)	123 (27.5)	125 (27.9)	*114* (*25.4)*		166 (37.1)	61 (13.6)	40 (8.9)	181 (40.4)*	
Workload, *n* (%)										
Increased	77 (16.2)*	118 (24.8)	148 (31.2)*	132 (27.8)*	<0.01	144 (30.3)*	79 (16.6)	39 (8.2)	213 (44.8)*	<0.01
Not increased	102 (31.7)*	100 (31.1)	66 (20.5)*	54 (16.8)*		175 (54.3)*	49 (15.2)	25 (7.8)	73 (22.7)*	

STAI Y1, State-Trait Anxiety Inventory Form Y1; IES-R, Impact of Event Scale – Revised.

* Statistically significant.

**Table 4:** Severity categories of evaluated psychological outcomes: subgroups analysis

Characteristic	MBI-EE (category)			*P-*value	MBI-DP (category)			*P*-value	MBI-PA (category)			*P*-value
	Low	Medium	High		Low	Medium	High		Low	Medium	High	
Overall, *n* (%)	297 (37.3)	176 (22.1)	324 (40.7)		346 (43.4)	210 (26.3)	241 (30.2)		290 (36.4)	222 (27.9)	285 (35.8)	–
Profession, *n* (%)												
Physician	121 (36.9)	70 (21.3)	137 (41.8)	Not significant	128 (39)	93 (28.4)	107 (32.6)	Not significant	113 (34.5)	98 (29.9)	117 (35.7)	Not significant
Nurse	176 (37.5)	106 (22.6)	187 (39.9)		218 (46.5)	117 (24.9)	134 (28.6)		177 (37.7)	124 (26.4)	168 (35.8)	
Gender, *n* (%)												
Male	85 (42.9)	36 (18.2)	77 (38.9)	Not significant	62 (31.3)*	59 (29.8)	77 (38.9)*	<0.01	66 (33.3)	55 (27.8)	77 (38.9)	Not significant
Female	212 (35.4)	140 (23.4)	247 (41.2)		284 (47.4)*	151 (25.2)	164 (27.4)*		224 (37.4)	167 (27.9)	208 (34.7)	
COVID-19 test, *n* (%)												
Negative	263 (37)	160 (22.5)	288 (40.5)	Not significant	317 (44.6)	182 (25.6)	212 (29.8)	Not significant	264 (37.1)	192 (27)	255 (35.9)	Not significant
Positive	34 (39.5)	16 (18.6)	36 (41.9)		29 (33.7)	28 (32.6)	29 (33.7)		26 (30.2)	30 (34.9)	30 (34.9)	
Children, *n* (%)												
Yes	199 (39.3)	109 (21.5)	198 (39.1)	Not significant	243 (48)*	138 (27.3)	125 (24.7%)*	<0.01	195 (38.5)	133 (26.3)	178 (35.2)	Not significant
No	98 (33.7)	67 (23)	126 (43.3)		103 (35.3)*	72 (24.7)	116 (39.9)*		95 (32.6)	89 (30.6)	107 (36.8)	
Living condition, *n* (%)												
With partner	216 (38.3)	125 (22.2)	223 (39.5)	Not significant	251 (44.5)	154 (27.3)	159 (28.2)	Not significant	217 (38.5)	146 (25.9)	201 (35.6)	Not significant
Without partner	81 (34.8)	51 (21.9)	101 (43.3)		95 (40.8)	56 (24)	82 (35.2)		73 (31.3)	76 (32.6)	84 (36.1)	
Family division, *n* (%)												
Yes	54 (26.2)*	55 (26.7)	97 (47.1)*	<0.01	72 (35)*	63 (30.6)	71 (34.5)*	0.02	73 (35.4)	53 (25.7)	80 (38.8)	Not significant
No	243 (41.1)*	121 (20.5)	227 (38.4)*		274 (46.4)*	147 (24.9)	170 (28.8)*		217 (36.7)	169 (28.6)	205 (34.7)	
COVID-19 contact, *n* (%)												
Rare	107 (45.7)*	51 (21.8)	76 (32.5)*	<0.01	134 (57.3)*	51 (21.8)	49 (20.9)*	<0.01	101 (43.2)*	61 (26.1)	72 (30.8)*	0.03
Frequent	190 (33.7)*	125 (22.2)	248 (44)*		212 (37.7)*	159 (28.2)	192 (34.1)*		189 (33.6)*	161 (28.6)	213 (37.8)*	
Job changes, *n* (%)												
No	149 (42.7)*	72 (20.6)	128 (36.7)*	0.02	159 (45.6)	93 (26.6)	97 (27.8)	Not significant	146 (41.8)*	94 (26.9)	109 (31.2)*	0.01
Yes	148 (33)*	104 (23.2)	196 (43.8)*		187 (41.7)	117 (26.1)	144 (32.1)		144 (32.1)*	128 (28.6)	176 (39.3)*	
Workload, *n* (%)												
Increased	140 (29.5)*	111 (23.4)	224 (47.2)*	<0.01	200 (42.1)	117 (24.6)	158 (33.3)	Not significant	156 (32.8)*	137 (28.8)	182 (38.3)*	0.04
Not increased	157 (48.8)*	65 (20.2)	100 (31.1.)*		146 (45.3)	93 (28.9)	83 (25.8)		134 (41.6)*	85 (26.4)	103 (32)*	

MBI-EE, Maslach Burnout Inventory – emotional exhaustion; MBI-DP, Maslach Burnout Inventory – depersonalisation; MBI-PA, Maslach Burnout Inventory – personal accomplishment.

* Statistically significant.

After correcting for confounders, multivariable logistic regression analysis showed that family division and increased workload were both independently associated with moderate-to-severe symptoms of state anxiety (odds ratio 1.96, 95% CI 1.3–2.9, *P* < 0.01; and odds ratio 2.9, 95% CI 2–4.2, *P* < 0.01, respectively), distress (odds ratio 2.14, 95% CI 1.5–3.1, *P* < 0.01; and odds ratio 2.35, 95% CI 1.7–3.3, *P* < 0.01) and emotional exhaustion (odds ratio 1.83, 95% CI 1.3–2.7, *P* < 0.01; and odds ratio 2.11, 95% CI 1.5–2.9, *P* < 0.01), as described in Table 5. Job changes were related with more moderate-to-severe symptoms of distress and emotional exhaustion (odds ratio 1.63, 95% CI 1.2–2.3, *P* < 0.01; and odds ratio 1.41, 95% CI 1.1–1.9, *P* = 0.03, respectively), whereas depersonalisation was higher for frequent contact with patients with COVID-19 (odds ratio 2.05, 95% CI 1.5–2.9, *P* < 0.01). Finally, HCWs presenting with trait anxiety was strongly associated with moderate-to-severe symptoms of state anxiety (odds ratio 8, 95% CI 5.7–11.3, *P* < 0.01) and distress (odds ratio 5.79, 95% CI 4.1–8.2, *P* < 0.01), but medium-to-high symptoms of emotional exhaustion (odds ratio 3.32, 95% CI 2.4–4.6, *P* < 0.01) and depersonalisation (odds ratio 2.44, 95% CI 1.8–3.3, *P* < 0.01). Trait anxiety was also associated with lower risk of having medium-to-low personal accomplishment (odds ratio 0.51, 95% CI 0.4–0.7, *P* < 0.01); after severity stratification, this relationship was only present for mild and moderate levels of trait anxiety (mild: odds ratio 0.51, 95% CI 0.4–0.7, *P* < 0.01; moderate: odds ratio 0.50, 95% CI 0.3–0.8, *P* < 0.01).

**Table 5:** Multivariate logistic regression analysis: risk factors for worst psychological outcome

Characteristic	Psychological scale, adjusted odds ratio for moderate-to-severe case (95% CI) and *P*-value									
	STAI Y1	IES-R	MBI-EE	MBI-DP	MBI-PA
Gender										
Male	Reference	*P* = 0.02	Not significant		Not significant		Reference	*P* <0.01	Not significant	
Female	1.61 (1.1–2.4)						0.46 (0.3–0.7)			
Children										
No	Not significant	Reference	*P =* 0.03		Not significant		Reference	*P* <0.01	Not significant	
Yes		1.47 (1.1–2.1)					0.62 (0.5–0.8)			
Family division										
No	Reference	*P* <0.01	Reference	*P* <0.01	Reference	*P* <0.01	Not significant		Not significant	
Yes	1.96 (1.3–2.9)		2.14 (1.5–3.1)		1.83 (1.3–2.7)					
COVID-19 contact										
Rare	Not significant		Not significant		Not significant		Reference	*P* <0.01	Not significant	
Frequent							2.05 (1.5–2.9)			
Job changes										
No	Not significant		Reference	*P* <0.01	Reference	*P* = 0.03	Not significant		Not significant	
Yes			1.63 (1.2–2.3)		1.41 (1.1–1.9)					
Workload										
Not increased	Reference	*P* <0.01	Reference	*P* <0.01	Reference	*P* <0.01	Not significant		Not significant	
Increased	2.9 (2–4.2)		2.35 (1.7–3.3)		2.11 (1.5–2.9)					
STAI Y2										
Absent	Reference	*P* <0.01	Reference	*P* <0.01	Reference	*P* <0.01	Reference	*P* <0.01	Reference	*P* <0.01
Present	8 (5.7–11.3)		5.79 (4.1–8.2)		3.32 (2.4–4.6)		2.44 (1.8–3.3)		0.51 (0.4–0.7)	

Multivariate logistic regression analysis was performed with the independent and dependent variables reported below. Odd ratios were assigned for every risk factor, after further correction for age. Independent variables were gender, presence of children, family division, contact with patients with COVID-19, job changes, workload and STAI Y2 score. Dependent variables (worst psychological outcome) were scores on the STAI Y1 (moderate or severe symptoms), IES-R (moderate or severe distress), MBI-EE (medium or high emotional exhaustion), MBI-DP (medium or high depersonalisation) and MBI-PA (low or medium personal accomplishment).

STAI Y1, State-Trait Anxiety Inventory Form Y1; IES-R, Impact of Event Scale – Revised; MBI-EE, Maslach Burnout Inventory – emotional exhaustion; MBI-DP, Maslach Burnout Inventory – depersonalisation; MBI-PA, Maslach Burnout Inventory – personal accomplishment; STAI Y2, State-Trait Anxiety Inventory Form Y2.

## Discussion

This work provides reliable data on the psychological impact of the COVID-19 pandemic in a large sample of HCWs in North-West Italy. Results show a high prevalence of state anxiety, distress and burnout, particularly in their moderate-to-severe forms, and their strict association with the family- and work-related conditions induced by the pandemic. In this context, only limited data in small samples of Italian HCWs are available, highlighting the relevance of our findings.^6,7^

Regarding the prevalence of anxiety and distress, our findings are in line with those reported in the Asian populations for the COVID-19 pandemic and previous descriptions for severe acute respiratory syndrome (SARS) and Middle East respiratory syndrome (MERS), where a massive impact of these outbreaks on HCWs has been described.^8,10–12,28^

Nevertheless, the presented data are consistent because they refer to the first European nation overwhelmed by the contagion. On 1 May – the date of survey closure – Italy was the most compromised European country in terms of deaths,^29^ and Piedmont was the second region for the number of cases,^15^ confirming the elevated psychological burden for HCWs even in a different sociocultural environment with different healthcare systems.

HCWs are subject to developing burnout syndrome, and different levels of emotional exhaustion, depersonalisation and personal accomplishment have been reported.^5^ Average levels of emotional exhaustion and depersonalisation are described in Italian HCWs in their usual working conditions;^30^ however, the baseline levels of burnout depend on the characteristics of the target sample, as well as on the size of the sample and the subjects enrolled in the sample.^31–33^ Nevertheless, the extent of the impact of the COVID-19 pandemic on HCWs’ burnout levels is still unclear, requiring further studies with larger samples. Preliminary evidence reported a high prevalence of burnout in HCWs during the pandemic, at around 50% in the three dimensions of burnout of emotional exhaustion, depersonalisation and personal accomplishment in several studies.^34,35^ Our findings confirm that moderate-to-severe levels of burnout are detectable in Italian HCWs facing the COVID-19 pandemic; contextually, they appear higher when referring to the normative values.^23^

Among professions, in line with previous evidence, nurses were more prone to develop severe distress compared with physicians.^12,36^ This finding was specifically driven by higher scores in avoidance and intrusion on the IES-R subscales in our sample. The connection between nurses and distress could be related to a higher susceptibility to infection because of the closer and more continuous relationship with patients. Thus, nurses’ vulnerability should be considered when implementing preventive measures, so as to protect them from psychological damage during a pandemic.^37^

Regarding gender, women appeared to be more prone to develop symptoms related to anxiety and distress, whereas depersonalisation was more frequently associated with men (odds ratio 2.17, 95% CI 1.5–3.2, *P* < 0.01). Even if gender differences in burnout are uncertain – particularly in a pandemic context where limited data are available – a tendency for major depersonalisation in men has been previously described.^38^ However, further investigation is needed to confirm this finding.

In addition, our findings suggest that division of the family nucleus during the pandemic was associated with worst outcome for developing distress symptoms and emotional exhaustion. Participants who decided to live at home but separated from loved ones experienced more psychological symptoms, probably because the socio-familial dimension has a relevant role in sustaining an individual's quality of life, and it is known that social support (i.e. sharing feelings, discussing choices and searching for help when needed) is an important resource for psycho-physical health.^39^ Moreover, evidence that family support reduces the risk of mental health issues is well established.^40^ Social isolation can exacerbate the management of distress;^41^ therefore, the socio-familial dimension of HCWs’ quality of life could play a considerable role in their mental health.

With regard to work-related factors, the increased workload, having frequent contact with patients with COVID-19 and changes in job duties were associated with more severe distress and emotional exhaustion both in physicians and nurses, in line with previous reports.^6,11,12^ In a critical condition in which healthcare systems are at risk of collapsing, as with the COVID-19 pandemic, these features could have exacerbated the risk of developing occupational burnout. However, interestingly, the same occupational factors were also associated with significantly higher levels of elevated personal accomplishment (Tables 3 and 4). We suggest that despite the adverse psychological conditions, these job-related difficulties could have increased HCWs’ personal satisfaction and sense of fulfilment, especially when associated with contributing toward the health of the community in terms of saving lives and limiting the negative effects of the crisis. Moreover, HCWs have been defined by the social media as ‘heroes’ for their dedication and work.^42^ As a result, their sense of social responsibility could have been amplified by this attribution of importance to their profession.^43^

Our data show that trait anxiety was strongly associated with state anxiety, distress and burnout during the pandemic. However, HCWs with mild-to-moderate levels of trait anxiety also demonstrated a significantly lower probability of having low personal accomplishment. Anxiety represents a warning reaction to external stimulations, and in its normal state, it enhances the ability of the individual to resolve specific situations. This is particularly true when its levels remain subthreshold; in contrast, beyond its threshold, anxiety becomes harmful. It has been postulated that negative emotions such as anxiety could be beneficial for performance; indeed, this so-called ‘anxiety motivation’ could have facilitative properties in specific circumstances.^44^ Even if the pandemic context generated unfavourable working and family conditions, it could also have provided determination and dynamism, helping to reach a future target. This functional utility of anxiety could have led to higher accomplishment, irrespective of the high levels of distress and emotional exhaustion.

In conclusion, our work provides consistent data on the psychological impact of exposure to COVID-19 and stress-associated factors on HCWs in North-West Italy. Clinically significant symptoms of anxiety, distress and burnout emerged in the examined sample. Nurses and HCWs who experienced family division, increased workload, job changes and trait anxiety were most at risk of developing such symptoms, with some gender differences. These findings are relevant in helping to identify individuals at higher risk of developing psychological concerns during a pandemic-related health crisis. Based on previous research and findings from this study, protective interventions dedicated to HCWs (i.e. providing valid instrumental support, guaranteeing an adequate job-related turnover, using clear operative protocols and making psychological support services available) are needed for protection from clinically relevant symptoms. In this regard, implementing individualised paths to enhance HCWs’ resilience could be useful for reducing burnout levels.^45^ The detection of factors associated with worst psychological outcome may favour a tailored, preventive, organisational and psychological approach, representing a potential strength in counteracting the effects of future pandemics.

### Limitations

This study has some limitations. First, we collected data from a large sample of HCWs in Turin, Italy, but the results are specific to the involved area and may not necessarily be generalisable to other healthcare systems or territories with a different pandemic situation. Moreover, among HCWs who had frequent contact with patients with COVID-19, we were unable to stratify them by the level of intensity of care. Second, we recruited only some of the healthcare professionals of the hospitals involved, leading to potential selection bias. Third, the investigation was conceived as cross-sectional and survey-based, thus offering a static snapshot of a pandemic that is, in contrast, an evolving and dynamic situation. Because of its design, longitudinal follow-up is lacking. Finally, the psychological scales used were self-reported and not directly assessed by a mental health professional.

## Data Availability

The data supporting the findings of this study are available from the corresponding author, A.N., upon reasonable request.

## Supplementary material

For supplementary material accompanying this paper visit https://doi.org/10.1192/bjo.2020.161. click here to view supplementary material
