# Supplementary material for: COVID-19 pandemic-related anxiety, distress and burnout: prevalence and associated factors in healthcare workers of North-West Italy
Source: BJPsych Open. 2021 Jan 7;7(1):e27. doi: 10.1192/bjo.2020.161 (PMC7844147; doi:10.1192/bjo.2020.161)
Supplement: Supplementary file 1 [file S2056472420001611sup001.docx]

**SUPPLEMENTARY MATERIAL**

- Scores and severity categories for STAI-Y2.

| **Psychological scale** | | **Overall**  **(n. 797)** | **Healthcare workers**  **Physician (n. 328) Nurse (n. 469)** | | ***p-value*** |
| --- | --- | --- | --- | --- | --- |
| **STAI-Y2** | |  |  | |  |
| Total Scoring *[Median (IQR)]* | | 41 *(34-48)* | 40 *(33-47)* | 41 *(35-48)* | ***0.02*** |
| **Categories** | Normal *[n. (%)]* | 358 *(44.9)* | 159 *(48.5)* | 199 *(42.4)* | *NS* |
|  | Mild *[n. (%)]* | 280 *(35.1)* | 110 *(33.5)* | 170 *(36.2)* |  |
|  | Moderate *[n. (%)]* | 128 *(16.1)* | 50 *(15.2)* | 78 *(16.6)* |  |
|  | Severe *[n. (%)]* | 31 *(3.9)* | 9 *(2.7)* | 22 *(4.7)* |  |

STAI: State-Trait Anxiety Inventory; NS: Not Significant

- Severity categories for STAI-Y2: subgroups analysis

| **Characteristic** | **STAI-Y2 (category)** | | | | ***p-value*** |
| --- | --- | --- | --- | --- | --- |
|  | **Normal** | **Mild** | **Moderate** | **Severe** |  |
| **Overall** *[n. (%)]* | 358 (44.9) | 280 (35.1) | 128 (16.1) | 31 (3.9) | *-* |
| **Profession** *[n. (%)]* |  |  |  |  |  |
| Physician | 159 (48.5) | 110 (33.5) | 50 (15.2) | 9 (2.7) | *NS* |
| Nurse | 199 (42.4) | 170 (36.2) | 78(16.6) | 22 (4.7) |  |
| **Sex** *[n. (%)]* |  |  |  |  |  |
| Male | 124 (62.6)* | 50 (25.3)* | 19 (9.6)* | 5 (2.5) | ***< 0.01*** |
| Female | 234 (39.1)* | 230 (38.4)* | 109 (18.2)* | 26 (4.3) |  |
| **COVID-19 test** *[n. (%)]* |  |  |  |  |  |
| Negative | 320 (45) | 245 (34.5) | 118 (16.6) | 28 (3.9) | *NS* |
| Positive | 38 (44.2) | 35 (40.7) | 10 (11.6) | 3 (3.5) |  |
| **Children** *[n. (%)]* |  |  |  |  |  |
| Yes | 243 (48)* | 173 (34.2) | 80 (15.8) | 10 (2)* | ***< 0.01*** |
| No | 115 (39.5)* | 107 (36.8) | 48 (16.5) | 21 (7.2)* |  |
| **Living condition** *[n. (%)]* |  |  |  |  |  |
| With partner | 265 (47) | 185 (32.8) | 95 (16.8) | 19 (3.4) | *NS* |
| Without partner | 93 (39.9) | 95 (40.8) | 33 (14.2) | 12 (5.2) |  |
| **Family division** *[n. (%)]* |  |  |  |  |  |
| Yes | 86 (41.7) | 67 (32.5) | 41 (19.9) | *12 (5.8)* | *NS* |
| No | 272 (46) | 213 (36) | 87 (14.7) | *19 (3.2)* |  |
| **COVID-19 contact** *[n. (%)]* |  |  |  |  |  |
| Rare | 114 (48.7) | 74 (31.6) | 34 (14.5) | *12 (5.1)* | *NS* |
| Frequent | 244 (43.3) | 206 (36.6) | 94 (16.7) | *19 (3.4)* |  |
| **Job changes** *[n. (%)]* |  |  |  |  |  |
| No | 161 (46.1) | 125 (35.8) | 50 (14.3) | *13 (3.7)* | *NS* |
| Yes | 197 (44) | 155 (34.6) | 78 (17.4) | *18 (4)* |  |
| **Workload** *[n. (%)]* |  |  |  |  |  |
| Increased | 203 (42.7) | 168 (35.4) | 84 (17.7) | *20 (4.2)* | *NS* |
| Not increased | 155 (48.1) | 112 (34.8) | 44 (13.7) | *11 (3.4)* |  |

STAI: State-Trait Anxiety Inventory; NS: Not Significant; *Statistically significant
